# Welcome to a new year and a new OPEN ACCESS JBC!

**DOI:** 10.1016/j.jbc.2020.100199

**Published:** 2020-12-31

**Authors:** Lila M. Gierasch

We turn the calendar from 2020 to 2021 with great shared relief … what a year! Despite the many disruptions, we have not been sitting still at JBC. With a trumpet fanfare, we want to celebrate with you the momentous occasion of JBC (and its sister ASBMB publications JLR and MCP) becoming gold open access! Your work in JBC can now, as of 2021, be accessed, read, and appreciated by readers all over the globe with no obstacles. Moreover, we’ve made our 115-year archive free to read—all of it. This is big.

This big announcement is only fitting for our beloved journal; after all, JBC is big. JBC publishes research, commentary, and reviews across the spectrum of biological chemistry writ large. Its content is continually evolving as the science of biological chemistry gets redefined and experiences intersections with other fields owing to newly formulated questions and advances in methodology. Yet the overarching scope of JBC remains: all science seeking to understand in molecular depth the workings of biology.

JBC’s mission is to foster advances in science that fall under this overarching scope. We have made many changes to how JBC handles manuscripts to ease the pain of authors who submit (1), to ensure rapid and rigorous review (2), to keep errors out of the literature (3), and to enhance the visibility of papers published in JBC (4). We changed our publication policies so that authors keep ownership of their work and give the journal a license to publish their articles (1). We launched JBC Reviews to provide topical, informative, and stimulating presentations of current research areas (5). And more! Every one of the changes and innovations to JBC that the editors have spearheaded has had a single-minded purpose: to help advance the science of the biological chemistry community.

The move to open access is one more action taken to help advance your science—and we might point out, this is a big one! At what cost? Well, much lower than what many other journals are asking for the same impact. The article processing charge for JBC is only $2000 for ASBMB members and $2500 for nonmembers. As practicing scientists ourselves, we share your goal of focusing those hard-fought grant dollars on doing the research, not reporting it.

We’re so delighted that the content of JBC is available to everyone. Please join us in raising a glass to toast the end of 2020, the beginning of a new year, and the launching of an open access JBC!!!
